# Nutrition as Personalized Medicine against SARS-CoV-2 Infections: Clinical and Oncological Options with a Specific Female Groups Overview

**DOI:** 10.3390/ijms23169136

**Published:** 2022-08-15

**Authors:** Miriam Dellino, Eliano Cascardi, Marina Vinciguerra, Bruno Lamanna, Antonio Malvasi, Salvatore Scacco, Silvia Acquaviva, Vincenzo Pinto, Giovanni Di Vagno, Gennaro Cormio, Raffaele De Luca, Miria Lafranceschina, Gerardo Cazzato, Giuseppe Ingravallo, Eugenio Maiorano, Leonardo Resta, Antonella Daniele, Daniele La Forgia

**Affiliations:** 1 Department of Biomedical Sciences and Human Oncology, University of Bari, 70100 Bari, Italy; 2 Clinic of Obstetrics and Gynecology, “San Paolo” Hospital, 70123 Bari, Italy; 3 Department of Medical Sciences, University of Turin, 10124 Turin, Italy; 4 Pathology Unit, FPO-IRCCS Candiolo Cancer Institute, Str. Provinciale 142, Km 3.95, 10060 Candiolo, Italy; 5 Fetal Medicine Research Institute, King’s College Hospital, London SE5 9RS, UK; 6 Department of Basic Medical Sciences and Neurosciences, University of Bari “Aldo Moro”, 70121 Bari, Italy; 7 Gynecologic Oncology Unit, IRCCS Istituto Tumori Giovanni Paolo II, Department of Interdisciplinary Medicine (DIM), University of Bari “Aldo Moro”, 70121 Bari, Italy; 8 IRCCS Istituto Tumori Giovanni Paolo II, 70124 Bari, Italy; 9 Department of Emergency and Organ Transplantation, University of Bari “Aldo Moro”, 70121 Bari, Italy

**Keywords:** COVID-19, SARS-CoV-2, ketogenic diet, nutrition in pregnancy, microbiota, cancer, carcinoma, precision medicine, health promotion, metabolomics

## Abstract

Coronavirus disease 2019 (COVID-19) is a respiratory disease caused by severe acute respiratory syndrome coronavirus-2 (SARS-CoV-2). It is acknowledged that vulnerable people can suffer from mortal complications of COVID-19. Therefore, strengthening the immune system particularly in the most fragile people could help to protect them from infection. First, general nutritional status and food consumption patterns of everyone affect the effectiveness of each immune system. The effects of nutrition could impact the level of intestinal and genital microbiota, the adaptive immune system, and the innate immune system. Indeed, immune system cells and mediators, which are crucial to inflammatory reaction, are in the structures of fats, carbohydrates, and proteins and are activated through vitamins (vit) and minerals. Therefore, the association of malnutrition and infection could damage the immune response, reducing the immune cells and amplifying inflammatory mediators. Both amount and type of dietary fat impact on cytokine biology, that consequently assumes a crucial role in inflammatory disease. This review explores the power of nutrition in the immune response against COVID-19 infection, since a specific diet could modify the cytokine storm during the infection phase. This can be of vital importance in the most vulnerable subjects such as pregnant women or cancer patients to whom we have deemed it necessary to dedicate personalized indications.

## 1. Pathobiology and Metabolic Dysfunction of SARS-CoV-2

Coronavirus 2019 (COVID-19) has become a global threat with a mortality rate around 6% [1]. In adults, the clinical manifestations of COVID-19 may or may not appear for the entire duration of the incubation period (2–14 days) and beyond, or they may be severe. Children are less likely to develop symptomatic infections and are less prone to serious illness [2], although there are reports of children who have a disease called Multisystem Inflammatory Syndrome [3,4]. COVID-19 belongs to the beta family of Coronaviridae and is known for the spike protein used to hook and infect the host cell [5,6,7], conferring COVID-19 easy transmissivity and high pathogenicity, [8] allowing the virus to: (i) fuse cell–cell and RNA release, (ii) start the replication cycle, and (iii) spread further to infect more cells [9,10], ensuring greater transmissibility (R0 > 2). From the ultrastructural point of view, the virus, having a higher specificity than other viruses of the same family [11], binds to the host via the binding domains of the angiotensin 2 converting enzyme receptor (ACE2), expressed more on the luminal surface of type II alveolar epithelial cells [12], resulting in an increase in angiotensin-2 with relative increase in pulmonary vascular permeability and subsequent severe acute respiratory syndrome or multiorgan dysfunction [13]. This clinical picture is due to the presence of acute inflammation, mainly CD4 and CD8 positive T lymphomonocytes, responsible for the recall of cytokines (interleukins such as IL-1β, IL-2R, IL-6, as well as interferon and tumor necrosis factor) and chemokines such as CCL-2, CCL-3, and CCL-10, which determine the hyperinflammatory state in COVID-19-positive patients [14]. Following this process, there is therefore the desquamation of pneumocytes, the formation of fibrinoid exudates and pulmonary edema, and finally formation of hyaline membrane with consequent irreversible alveolar damage. Conversely, during the recovery phase, there is an increase in pro-coagulation factors and subsequent activation of the coagulation cascade with the formation of small and large peripheral vascular thrombi also known as disseminated intravascular coagulation [13,15]. In more critically ill patients, laboratory tests also show hyperferritinaemia, elevated lactate dehydrogenase, and erythrocyte sedimentation rate [16]. This pathological structure can also be associated with further complications at the olfactory level [17] or at the visual level (eye pain, redness, and conjunctivitis), splenic and hepatic level where drug toxicity and immune-mediated damage play a role [18], renal, neurological, and skin level such as erythematous rashes and urticaria, lympho-haematological [19,20,21,22,23,24,25,26], and even at the level of the cardiovascular system [27,28,29] where patients with SARS-CoV-2 have an increased risk of developing acute myocardial infarction following coronary spasm, hypoxic damage, microthrombi, direct vascular endothelial damage, hypercoagulability, and instability of the atherosclerotic plaque [30]. It is also known that the downregulation of ACE2 and the increased stimulation of the angiotensin II receptor are associated with systemic hypotension, hypokalaemia, and lung damage [31]. As is well known, iron represents one of the main elements in the fight against pathogens, given its ability to support the immune system during the viral replication phase. In fact, since high availability of iron is required for COVID-19 to allow the hydrolysis of the ATP required in this phase, the innate immune system will modify the bioavailability of iron downwards, limiting its ability to replicate and helping the body to fight the virus. Ferritin is the most important component of iron metabolism, and its main role is to store iron during the ferric state during infections. During the inflammatory phase, the serum iron concentration decreases and ferritin increases, causing hyperferritinemia and activation of macrophages. Regulating this mechanism is hepcidin, capable of regulating the concentration of intra- and extracellular iron thus depriving the pathogen of iron for the replication of COVID-19 [32,33]. The metabolic processes of iron are also generated by the generation of reactive oxygen species (ROS) [34], which can cause mitochondrial dysfunction favoring multiple-organ damage as occurs during COVID-19 infection [34,35]. Alterations at this level lead to an upregulation of mitochondrial genes and genes that respond to oxidative stress with further accumulations of intracellular iron. These generate ROS and reactive nitrogen and sulphur species, further contributing to the increase in the inflammatory response in COVID-19 disease. In fact, the same mitochondrial ROS also directly activates the production of proinflammatory cytokines that can alter mitochondrial homeostasis and mitochondrial respiration thus causing various systemic alterations [36]. The inflammatory phase also leads to a depletion of the body’s natural antioxidant agents such as vit C, which in favorable physiological conditions protects cells, decreases oxidative stress and ROS, and improves the body’s circulatory function and its immune system. In critical conditions, its concentration drops precipitously so as to require intravenous administration to reach a quantity sufficient for the body. Although still in need of robust confirmation, on this basis clinical studies have been conducted on the treatment of COVID-19-positive patients, which have shown how this supplement can improve the critical condition of some patients [37,38]. Similarly to vit C, vit D also has the same effect on oxidative stress and provides a protective effect from COVID-19 but, also in this case, further studies should be encouraged to shed light on the effects of vit D deficiency in COVID-19-positive patients [39,40,41].

## 2. Microbiota and SARS-CoV-2 Infection

**Figure 1:**
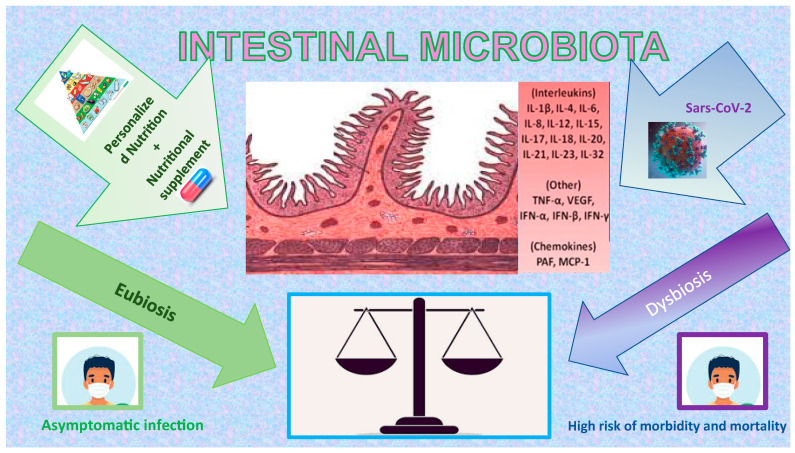
Impact of COVID-19, nutrition, and nutritional supplement on Intestinal Microbiota. The figure is a graphic illustration of how the correct use of macro- and micronutrients can be the ideal balance of our organism in the defence or rapid recovery to COVID-19, having a positive effect on the Intestinal Microbiota (green side), mainly for their antioxidant and anti-inflammatory properties.

Lately, different authors have reported in literature the possibility, more than suggestive, of a connection between COVID-19 infection and intestinal microbiota dysbiosis, considering that, in COVID-19 patients with severe intestinal dysfunction, the presence of virions in fecal, oral, and gastrointestinal samples was found in a range between 2% and 36% [42,43,44,45,46,47,48,49,50,51]. The most commonly reported intestinal symptoms related to COVID-19 could be mild as nausea and stomach discomfort or more intense such as vomiting and diarrhea [27,52,53,54,55]. Moreover, variations of the intestinal microbiota, with an increase in opportunistic pathogenic germs and reduction of protective commensal bacteria, relate to fecal levels of SARS-CoV-2 and severity of symptoms from COVID-19. Furthermore, this microbiota alteration could continue even after the eradication of SARS-CoV-2 and after the remission of disease symptoms [56]. Recent scientific evidence provided that the gravity of COVID-19 symptomatology is linked to comorbidity and advanced age since both aspects are connected to inflammation and alteration of the intestinal microbiota, in consideration of both the nature of the intestinal flora and its bacterial composition [57]. Consequently, it has been assumed that an intervention directed at supporting the intestinal barrier and reducing the inflammatory stimulus by recommending a diet rich in fiber and fermenting foods could be suitable to reduce the infection and gastrointestinal symptoms linked to COVID-19 [58]. Therefore, it is supposed that COVID-19 infection might be capable of modifying and undermining the integrity of the gut microbiota with a consequent higher gravity of the disease and complications [58]. Consequently, the intestinal biological components and its related dysbiosis are considered a potentially fundamental element to influence the adaptive response versus respiratory pathogens [59]. Indeed, recent studies have reported that patients with COVID-19 infection and underlying comorbidity with consequently reduced gut microbiota diversity and greater intestinal permeability showed a worse prognosis [52]. Confirmation of this evidence was the evaluation of how food microbes, such as probiotics or prebiotics, develop an antiviral effect against coronaviruses and could strengthen host immune functions. Moreover, a sensible decrease of Lactobacillus and Bifidobacterium species has been found in patients affected by COVID-19, even if the clinical meaning of this result is not yet defined [60]. Therefore, intestinal microbiota [61] could lead healthy subjects to an hyperinflammatory condition [62], which enhances the harmful effects of COVID-19. Indeed, composition of the intestinal microbiota is involved in the production of inflammatory cytokines and has an important role in the induction, development, and correct function of the host immune system and its activity [63]. In addition, ACE2 protein is widely distributed on the luminal surface of intestinal epithelial cell [64,65]. Indeed, recent evidence has reported that the “cytokine storm” may be the key that aggravates the morbidity and mortality of the COVID-19 infection [66] (Figure 1), and there is even evidence that COVID-19 affects women less and causes a stronger T-cell response than males, leading to more effective viral clearance [67,68,69].

This condition of hyperinflammation is also documented in histological specimens of the colon and mesentery [70]. Therefore, anti-cytokine therapy for reducing patients’ hyperinflammatory status could be recommended for the treatment of patients with severe COVID-19 and particularly in immunosuppressed subjects [71]. Furthermore, the prescription of a balanced diet, able at the same time to provide the organism with an adequate nutritional supply, can favor the correct functioning of the immune system [72]. In this regard, specific micronutrients such as vit (C and D) or elements such as zinc, taken directly from food through a correct diet or prescribed in the form of supplements, represent for this purpose a valid support having the dual function of guaranteeing a contribution to the organism for its physiological functions and allowing it to have a continuous reserve in pathological conditions, ensuring a constant and strong immune function even in critical situations [73]. Moreover, the recent literature is unable to provide data so concrete and reliable as to consider the clinical use of probiotics in patients with symptoms related to COVID-19, and therefore their administration cannot be officially recommended until there are experimental results that will be clearer on their relationship with the intestinal microbiota in COVID-19 patients, as well as on their functionality and usefulness in the fight against COVID-19 [74]. However, it is supposed that an approach that modulates the intestinal microbiota could represent one of the therapeutic approaches of COVID-19 and its complications [75]. This is especially important for our vulnerable populations, such as the elderly, and oncological and immunosuppressed patients, and it requires further, in-depth research. On the other hand, the role of the microbiota linked to COVID-19 has not yet been studied in newborns, but it is shown that through breastfeeding, vaccinated women [76,77,78,79,80,81,82] confer immunity to their children; an increase in IgG and IgA antibodies against SARS-CoV-2 [83,84,85,86] and consequent decrease in the risk of hospitalization was demonstrated [87,88]. Preliminary studies on these positive effects had concerned the analysis of these antibodies also at the level of the umbilical cord and placenta [89,90,91]. This research represents a driving force for future studies in this patient class.

## 3. Nutrition in Pregnancy during COVID-19 Infection

Pregnancy represents a special biological phase of the life of a woman and determines a lot of indicatory physiological changes involving all organ systems in the body with the main aim to sustain the growing fetus [92]. Nutrition represents a pivotal point for the physiological changes of pregnancy and, in this regard, childbearing causes an increased water need in order to expand maternal blood water and increase cardiac output from 4 to 6 L/min and, thus, blood flow to the kidneys and utero-placental unit, decreasing blood pressure overall [93]. The increase in tidal volume requires an average increase in oxygen demand in pregnancy of about 20% of minute ventilation. Breathing and nutrition are closely related processes. Through the digestive system, oxygen is used to oxidize the nutrients obtained from ingested foods and to obtain the necessary energy. Physiological changes in respiratory function in pregnancy allow the umbilical vein blood amount to be rich enough in oxygen for the fetal cellular respiration process [94]. Nutrition means fetal growth; especially in the third trimester of pregnancy, hormones of placental origin determine a state of insulin resistance in the pregnant woman; a condition of hyperglycaemia is established, which maintains fetal growth and lipolysis to satisfy maternal needs [95]. The mother’s requirement for increased insulin could be a biological solution to promote fetal growth, while insulin itself can remain the main growth factor alongside conception product development [96]. It is known that nutrition plays an essential role in the development and maintenance of the immune system. Its deficiency can negatively affect the risk of infections and the maternal organism’s acceptance of the product of conception, characterized by different genetic and haplotypic heritages [97]. If innate immunity (NK cells and monocytes) is preserved, acquired immunity (T cells and B cells) is downregulated, harming any specific immune response, hence causing an immunosuppressed state [98]. In addition, the inflammatory response differs alongside pregnancy according to the three main stages: the pro-inflammatory stage (implantation and placentation in the first trimester), the anti-inflammatory stage (fetal growth in the second trimester), and the second pro-inflammatory stage (initiation of parturition) [99]. Intuitively, nutritional deficiencies can compromise the immune response by greatly interfering with the body’s response to external inputs, leading to an amplification of the pathophysiological pathways and, thus, conditioning the corresponding outcomes [92]. To date, the SARS-CoV-2 infection represents one of the most interesting examples of how peculiar the interactions of the systemic range of the action pathogen are, i.e., a peculiar biological environment [97]. From this point of view, nutrition might act as a synergic co-factor, which may be able to influence organism response, while taking into consideration its significance is still under scientific evaluation. It is established that macronutrient intake variably influences the immune system response and inflammatory pathways. Thus, it may be used as a predictive factor for response to SARS-CoV-2 in pregnant women. To date, only a few papers are available. Moreover, these existing papers (Table 1) might be used to discriminate between food and macronutrients useful or harmful in COVID-19 infection management [100].

The need of FA is not the same for the whole population and reaches its maximum levels during pregnancy and breastfeeding. Since the 1990s, in order to prevent NTDs (neural tubal defects), a daily intake of FA (folic acid) between 400 and 600 mcgr has been highly recommended during pregnancy by most health systems around the world; this is to compensate the insufficient average dietary intake of FA. A nutrition that includes fresh fruit and vegetables is therefore essential to meet the daily requirements of folate. FA also contributes to the physiological anemia of pregnancy, promoting hematopoiesis and therefore the increase in Hb levels [105]. This works only if there is enough iron deposited to be used. In a case-control study during the COVID-19 pandemic, the authors [104] demonstrated that it is mandatory to screen for and treat anemia with both FA and iron. They found that among pregnant women with iron deficiency anemia, the COVID-19 group had a higher risk of puerperal infection, emergency c-section and SGA (small for gestational age). Low birth weight, prematurity, and lower APGAR scores were also more frequent in the COVID-19 group. Specifically, a daily combination of FA and iron could help to normalize weight of the newborn in this setting [104]. According to the latest international guidelines, it is mandatory to increase the daily FA dose up to 4–5 mg in case of: previous NTD-affected pregnancy, neurological disease and malformations, contemporary therapy with anti-epileptic drugs, or, with malabsorption disease and high maternal BMI and/or impaired stages of glucose metabolism. This latter group might be more vulnerable to contracting the COVID-19 viral infection, as reported in (Eskenazy et. al., the INTERCOVID Study, 2021). According to this study, GDM (gestational diabetes mellitus), pre-existing DM, and being overweight or obese are all independent risk factors for SARS-CoV-2. An appropriate low-calorie dietary regimen besides eventual pharmacotherapy is needed for weight control during pregnancy and to improve pregnant women’s immune-receptivity. Indeed, in the DM and GDM groups, the obese and overweight women were at a higher risk of having COVID-19 than the normal weight women, and also the insulin-dependent diabetes group was at higher risk of COVID-19 than the diet-therapy group both for DM and GDM [95]. Mate et. al. emphasized the role of a well-balanced dietary regimen for pregnant woman in the SARS-CoV-2 setting, while focusing on the main nutrient useful to boost the immune system against systemic viral infection [100,106]. It should be mandatory to adopt an adequate nutrition and eventual feeding supplement not only during pregnancy, but also from preconception to the early post-natal period. The model of “food safety” which has been proposed is based on the experience of other viral and/or respiratory infections during pregnancy, which are well-known in the relevant literature. The core concept is to base the dietary regimen on functional foods, i.e., fruit and vegetables, including sufficient amounts of recommended micronutrients such as vit A, B, D, E, Omega-3 Fatty Acids, Choline, Zn, Fe, Se, and, as mentioned above, FA. This helps the immune system and has a positive impact on pregnancy outcomes in case of viral infection, including SARS-CoV-2. Omega-3 poly-unsaturated fatty acids down-regulate the excessive inflammatory response triggered by some systemic viral infections. Choline, in the case of respiratory virus infections, improves adverse fetal effects. The main adverse effects linked to not recommended food during pregnancy in case of systemic infection are reported in the existing literature: IUGR (intrauterine growth restriction), increased infant mortality, congenital diseases, miscarriage, preeclampsia, and nervous system anomalies or dysfunction [100]. It is important to mention the national cross-sectional study conducted in China by Chen et al. in 2022 on the sample of 3678 pregnant women during the COVID-19 pandemic. The Authors reported that a median daily intake of so-called functional food, like vegetables and fruit, significantly decreased to low, moderate, and high severity of pandemic, and, in this sense making immune system more vulnerable. In addition to the low quality of the foods consumed, the perinatal outcomes got worse [101]. Interestingly, researchers in Northern New England have proposed a model to identify and address questionable food choices among pregnant women during the pandemic period, highlighting the need for specific food programs [107]. Erol et al. discussed that vit E and Afamin are significant predictors of adverse perinatal outcomes in COVID-19 infected women. Specifically, vit E levels [102] were significantly lower than in the healthy group of women. In addition, Afamin levels were significantly higher, while positively correlated with CRP (C reactive protein) levels. The higher adverse perinatal outcomes in COVID-19 groups are due to higher oxidative stress [102]. A subsequent study conducted by Erol et al., 2021 enlightened readers on the possible correlation between maternal selenium status and clinical outcomes of pregnant women with the SARS-CoV-2 infection. They found that selenium levels negatively correlate with gestational week, D-dimer, and interleukin-6 (IL-6). As the infection got worse, researchers could observe lower selenium levels and higher inflammatory factors. This vicious circle leads to a higher maternal need for selenium, and it makes a selenium supplement mandatory [102]. According to the study conducted by Anuk et al. on pregnant women affected by SARS-CoV-2, serum zinc levels also negatively correlate with IL-6, and with other acute phase markers, i.e., erythrocyte sedimentation rate, PCT (procalcitonin), and CRP [103]. On the other hand, the serum copper level showed a positive correlation. Therefore, serum Zn/Cu levels were a reliable predictor of viral infection severity in pregnancy. Similarly, in the case when the increased serum magnesium levels were found, there were predictors of pancytopenia and higher CPR [103].

The appropriate assessment of the nutritional status of pregnant patients during and in post-COVID-19, is one of the pillars in the management of these patients. On the other hand, personalized dietary recommendations for these patients represent the best strategy to ensure their recovery. Unfortunately, data on SARS-CoV-2 and nutrition during pregnancy are still in the process of collection, and models are in the developing phase. However, a recommended feeding protocol should be developed in the future.

## 4. Nutrition in Oncologic Patients during COVID-19 Infection

The different female carcinomas [108,109,110,111,112,113,114,115,116,117,118] can influence nutritional status, with weight loss seen in 30–80% of early cases (observed in the 6 months prior to diagnosis) and correlated with the stage of the disease. Weight loss and malnutrition are the result of a reduction in food intake, in the presence of symptoms such as mycosis of the oral cavity, anorexia, nausea, vomiting, dysphagia, and dysgeusia related both to the site of the tumor and to specific treatments; undernutrition and loss of weight and muscle mass can lead to a greater risk of chemotherapy toxicity, while in other cases anticancer therapies and chemotherapy can lead to weight gain resulting in excess malnutrition, and then overweight/obesity, which represent risk factors for metabolic syndrome. In these patients with a compromised nutritional status, COVID-19 infection can further worsen the prognosis as the virus acts on the nutritional status by producing hypercatabolism and rapid muscle wasting [119]. The leading cause of death among infected patients is basically an acute respiratory distress syndrome caused by generalized inflammation. Several studies have analyzed the nutritional status and mortality among cancer patients diagnosed with COVID-19, demonstrating that some immune inflammatory parameters such as the number of lymphocytes could be associated with lower survival. Among other factors, being overweight/obese contributes to the overproduction of proinflammatory IL-6 and reduces cytotoxicity of Natural Killer cells [120]. The assessment of nutritional status in these patients should also take into consideration the possibility of sarcopenia development. The decrease in concentration of some proteins present in the serum such as albumin is often associated with malnutrition, inflammation, and lymphopenia that are negative prognostic factors in these patients [121]. Sarcopenia, defined as the depletion of skeletal muscle mass and muscle strength, was associated with the proliferation of peripheral mononuclear cells, and damaged homeostasis of natural killer lymphocytes [122]; the main mechanism underlying impaired immunity refers the abnormal myokines such as interleukin IL-6, IL-15, and IL-17 [123]. Regarding metabolic stress during severe infection by COVID-19, skeletal muscle is catabolized to provide the immune system, liver, and gut with amino acids [124]. In light of the above, the nutritional management of these patients is essential. Evaluation is a priority in order to be able to assign a correct nutritional therapy considering that these oncological patients affected by COVID-19 have an increased protein-caloric requirement due to the infection and fever. In a recent study of 2021, Sukkar SG et al. showed that the administration of a ketogenic diet characterized by a low carbohydrate (20–120 g) content, high lipid content, and normoprotein in patients with COVID-19 results in a reduction in mortality and artificial ventilation in these subjects. Ketosis protects healthy tissues against oxidative stress as it decreased production of reactive oxygen species and increased endogenous antioxidant capacity. The ketogenic diet can also inhibit inflammation, reducing circulating inflammatory markers. A KD, via hydroxybutyrate, is capable of activating hydroxycarboxylic acid receptor 2, which inhibits nuclear factor kb in macrophages, dendritic cells, and microglia reducing neuroinflammation [125]. As part of a ketogenic diet, it is possible to combine an intermittent fasting regime that many authors have proposed to alleviate the symptoms in the course of COVID-19 infection. Intermittent fasting is a practice that involves the restriction of eating and there are several approaches. For example, with 16/8, food is consumed only in 8 h or the 5:2 approach, which involves eating regularly five days a week and limiting daily calorie intake on the other two days, to 500–600 calories; it has gained popularity in recent years and shows promise as a possible new paradigm in the approach to weight loss and the reduction of inflammation and has many potential long term health benefits. There is good evidence that IF can benefit cardiometabolic health by decreasing blood pressure, insulin resistance, and oxidative stress. According to a study published in BMJ Nutrition, Prevention & Health, intermittent fasting could reduce the risk of hospitalization or death in patients who have contracted COVID-19 [126]. A metabolic change in IF is the increase in linoleic acid-enriched triacylglycerol species in the liver and serum during fasting. It has been hypothesized that linoleic acid (a polyunsaturated fatty acid of the omega 6) locks the spike protein of SARS-CoV-2 in a confirmation that is not conducive to the effective binding to ACE2. Besides elevated concentration of linoleic acid during fasting, it may lessen the number of infected cells and the number of SARS-CoV-2 virions in cells and so decrease the severity of the symptoms. Another benefit of IF is the increased levels of galectin-3, and this protein has been shown to bind directly to pathogens and have various effects on the functions of immune cells. Particularly, a randomized human study of low-frequency (once-per week) 24-h water-only intermittent fasting showed that fasting increased galectin-3 level over a moderate term and this protein involved in host defence to infectious diseases [127,128] in that it stimulates anti-inflammatory effects by modulating nuclear factor kappa-light-chain-enhancer of activated B cells (NF-κB) and the NLR family pyrin domain containing 3 (NLRP3) inflammasome [129], which should inhibit the hyper-inflammation associated with COVID-19. On the others hand, a number of micronutrients, including vitamin D, zinc, and omega-3 fatty acids have been shown to play key roles in supporting immune function [130,131,132] and in reducing risk of respiratory infection [131]. These nutrients can be obtained from the diet and are available as dietary supplements either alone or as part of multivitamin or multi-nutrient mixtures. A biologically plausible role exists for certain vitamins and minerals in immune pathways. Vitamin D is a steroid hormone involved in the modulation of the innate and acquired immune system as well as in the production of the antimicrobial peptides such as human β-defensin-2 and cathelicidin, in addition to the expression of genes responsible for the destruction of the intracellular pathogens. Many studies have consistently suggested that vitamin D deficiency is associated with increased risk of respiratory tract infections, especially in influenza and now COVID-19 [133,134,135,136], and some studies have suggested that it reduce SARS-CoV-2 transmission by enhancing antiviral immunity and to reduce mortality by mitigating the cytokine storm linked with severe COVID-19 [137]. Zinc is a trace element with potent immunoregulatory and antiviral properties and is utilized in the treatment of COVID-19. In a study in 2020, Jothimani D. et al. demonstrated that zinc deficient patients developed more complications, and the deficiency was associated with a prolonged hospital stay and increased mortality [138]. Other protective elements against infection have been hypothesized to be the very-long chain omega-3 fatty acids (EPA and DHA) that have anti-inflammatory properties that may help reduce morbidity and mortality from COVID-19 infection. These omega-3 s are associated with lower levels of circulating inflammatory cytokines, and intervention with fish oils reduces levels. EPA and DHA are precursors to a suite of inflammation-resolving mediators (IRMs [139]) that actively regulate the resolution of acute inflammation. IRMs down-regulate cytokine production and promote a return to homeostasis via monocyte/macrophage-mediated uptake of debris, apoptosis of neutrophils, and clearing of microbes. Accordingly, higher intakes of EPA and DHA (which result in higher RBC EPA+DHA levels, hereafter called the Omega-3 Index, O3I, have been proposed to lower the risk for adverse outcomes from COVID-19 [140,141,142,143,144,145,146], and case reports suggesting benefit have been published. An excessive inflammatory response, called a ‘cytokine storm’, is a frequent occurrence of the severe form of COVID-19. Omega-3 fatty acids have potent anti-inflammatory activities, and these fatty acids can mitigate the cytokine storm of COVID-19 such as suggested in a study pilot by Arash Asher et al. [147]. Maintaining a good nutritional status is essential for the functioning of the immune system and for defence against viral diseases. According to the European Society for Clinical nutrition and Metabolism (ESPEN) guidelines of good nutrition in patients with SARS-CoV-2, it depends on an adequate level of intake of nutrients such as vit D, A, C, B, and zinc, copper, iron, and selenium, which play a key role in the prevention of malnutrition [148].

## 5. Discussion and Potential Future Approaches

For about two years, the main symptom investigated by the vast majority of world researchers in the fight against COVID-19 was pulmonary [149,150,151,152,153,154]. Effort was due first of all to the impossibility of knowing how to manage a pandemic with the health complications that have arisen and, secondly, to the absence of targeted COVID-19 drugs, which in the first pandemic phase saw the experimentation of therapies already existing for others purposes and, in principle, were at least adaptable to the pulmonary and systemic symptoms presented by patients COVID-19 affected [155,156,157,158,159,160]. The advent of vaccines [85,161,162,163] has in fact revolutionized this perspective as they have guaranteed not only a preventive and protective effect in the contagion phase, but also a therapeutic effect as severe symptoms have increasingly given way to milder symptoms or even a lack of symptoms in the vaccinated population. This aspect must also be taken into consideration for extra-pulmonary symptoms, even in the long-term, assuming future viral variants and future vaccines. Proof of this is the study by Ran Barzilay et al., which, in a cohort of 3000 patients, showed that extrapulmonary symptoms were reduced by more than 50% in vaccinated patients, highlighting how vaccinated subjects have lower risks than unvaccinated ones [164]. Preliminary data from other studies go in this direction, reinforcing the concept that vaccination tends to reduce the most common symptoms related to COVID-19 [165]. Comparisons between these two classes also showed that on average there is a higher likelihood of having no symptoms among vaccinated than unvaccinated and reversed when considering the total number of symptoms or hospitalization [166,167]. For the future, supplementary comparative studies between these two classes would be desirable in relation also to the use of micronutrients, in addition to those specific to the gender inherent in the adverse effects of vaccination. In this sense, the data are in fact discordant considering that some authors report comparable data between males and females, while others do not, also in relation to the side effects after the first dose of the vaccine [168,169,170,171].

Probably today one of the least investigated aspects of the pathogenesis and development of COVID-19 manifestations is related to nutrition and how this can positively influence both in the short and long term. This topic is therefore both current and of great social impact considering that both physical and psychological manifestations related to COVID-19 have been identified, which can persist long-term even beyond recovery [172]. In fact, several authors around the world have also reported symptoms of psychological stress such as: sleep loss, nervousness, fatigue, depression, anxiety, [173,174,175,176,177] especially in the female population, very often linked to other cofactors such as the social and economic status or the patient’s health. Fortunately, these are often of a transitory type, especially if considered short-term [178], but, in the literature, there are several manuscripts that [172,179,180,181,182,183] report an up to three times increased risk in women of experiencing long-term symptoms. Unfortunately, on these analyses, there does not seem to be uniformity of results as other authors report discordant data [184,185,186]. These discrepancies suggest that additional factors, perhaps little studied to date, may contribute to these mechanisms. In fact, ethnic or hormonal causes have been seldom investigated in women with COVID-19. The latter, in fact, seem to influence the state of hyperinflammation even after healing [187,188] and consequently on the production of IgG antibodies [189]. These studies highlight some critical issues and consequently some perspectives still to be explored on COVID-19. The first of these is linked to the impossibility of having long or very long-term studies, capable of evaluating the effects of the COVID-19 infection in a prolonged manner; this lack may be the reason for conflicting data, which could in part be filled by a wider sharing of data by different research groups through the development of global rather than national studies or single research groups. This effort could actually confirm or dispel contributing causes such as ethnicity or the role of hormones in long-term effects. New confirmations on the role of gender could imply targeted therapeutic solutions or personalized prevention models; targeted research looking in this direction would therefore be desirable [190]. Another important aspect that deserves more study is the evaluation of long-term effects in patients with already established chronic diseases such as diabetes, arterial hypertension, or pulmonary dysfunction. In these pathologies, patients very often present with immune system alterations and an increase in pro-inflammatory factors, which together with COVID-19 could exacerbate the immune response or delay its effectiveness, resulting in slower or more difficult healing. Food supplementation could represent a valid support in these patients, especially if in old age or with slower recovery capacity, as already evaluated by some authors but only in a short time [179,182]. This aspect is even more important in those patients who, being bedridden for very long periods, risk going into sarcopenia; dietary implementation with the use of food supplements, or probiotics such as creatine or phosphocreatine could be an important aid in slowing down muscle resorption and facilitating its anabolism in this class of patients. Moreover, it is possible that this dietary approach may be useful in association with anticancer drugs such as Bevacizumab [117], used in the fight against COVID-19 as antiangiogenics for its treatment [37,191]. It is also no coincidence that, albeit slowly, the first studies on natural antioxidants such as vit C or D begin to appear in the literature, which demonstrate an improvement in critically ill patients by supporting their immune system, even if, in this regard, data should be more robust to be widely used in routine clinical practice [38,40]. Another possible aspect to be investigated with particular interest could be inherent to hepcidin agonists that can be used to increase endogenous production, allowing their use to decrease ferroportin and iron levels [192]. Furthermore, natural hepcidin inducers are also present in fruits and vegetables [193]. Finally, interesting approaches could also be described in the future regarding the relationship between COVID-19, nutrition, and changes in the intestinal and vaginal microbiota [118].

## 6. Conclusions

In recent years, COVID-19 and the resulting global pandemic have raised many both direct and indirect issues. To date, global efforts have been directed not only at improving vaccines against the virus but also at developing ever more targeted and specific medical therapies. It is therefore evident that the study of the mechanisms of action of the virus but above all of the excipients or supplements that can interact with the viral inflammatory process is a fundamental pillar in the fight against COVID-19. From this point of view, diet, through the intake of vit and micronutrients, represents the first bulwark in the prevention and fight against the virus and its associated symptoms, necessary for hospitalization or not. Furthermore, nutrition takes on the character of personalized medicine based on the patient’s condition. This is more significant in women where one must also consider other possible factors than the normal physiological or oncological state, such as pregnancy. Based on these assessments, the characteristics and needs of the immune system change together with the ability to react to the infection and regulate its spread at a multi-organ level. This highlights how diet can change and be reshaped as needed through assessments and personalized indications based on the state of health of COVID-19 positive patients.

## Figures and Tables

**Table 1:** All the studies existing to date investigating the effects of specific diet regimen, foods, micro-, or macronutrients on the response to COVID-19 viral infection in pregnant women.

Paper ID	Year of Publication	Type of Study	Cohort (*n*°)	Diet Regimen	COVID-19 Outcomes
Eskenzi et al. [95]	2021	Prospective, multinational	2071	Low-calorie diet in glucose altered metabolism or overweight	Improving
Chen et al. [101]	2022	Prospective, nationwide (China), multicenter	3678	Diet based on vegetables and fruit	Improving
Erol et al. [102]	2021	Prospective case-control (Turkey)	60	Lower vit E Higher Afamin serum levels	Worsening Worsening
Anuk et al. [103]	2021	Prospective case-control (Turkey)	200	Lower Zinc Higher Copper Lower Zn/Cu ratio Higher Magnesium serum levels	Worsening Worsening Worsening Worsening
Uta et al. [104]	2022	Prospective case-control (Romania)	446	Iron deficiency Iron and folate supplement (in anemic women)	Worsening Improving
Nawsherwan et al. [92]	2020	Review	-	Micronutrient deficiency and/or low intake (vit A, C, D, E, Fe, Se, Zn)	Worsening
Mate et al. [100]	2021	Review	-	“Functional food” diet (vegetables, omega3 fatty acid, micronutrient, vit A, B, D, E, choline)	Improving

## Data Availability

Not applicable.
